# UBASH3A deficiency accelerates type 1 diabetes development and enhances salivary gland inflammation in NOD mice

**DOI:** 10.1038/s41598-020-68956-6

**Published:** 2020-07-21

**Authors:** Yi-Guang Chen, Ashley E. Ciecko, Shamim Khaja, Michael Grzybowski, Aron M. Geurts, Scott M. Lieberman

**Affiliations:** 1 Department of Pediatrics, Medical College of Wisconsin, Milwaukee, WI 53226 USA; 2 Max McGee National Research Center for Juvenile Diabetes, Medical College of Wisconsin, Milwaukee, WI 53226 USA; 3 Department of Microbiology and Immunology, Medical College of Wisconsin, Milwaukee, WI 53226 USA; 4 Department of Physiology, Medical College of Wisconsin, Milwaukee, WI 53226 USA; 5 Cardiovascular Center, Medical College of Wisconsin, Milwaukee, WI 53226 USA; 6 Stead Family Department of Pediatrics, Carver College of Medicine, University of Iowa, Iowa City, IA 52242 USA

**Keywords:** Immunology, Autoimmunity, Immunogenetics

## Abstract

Recent advances in genetic analyses have significantly refined human type 1 diabetes (T1D) associated loci. The goal of such effort is to identify the causal genes and have a complete understanding of the molecular pathways that independently or interactively influence cellular processes leading to the destruction of insulin producing pancreatic β cells. *UBASH3A* has been suggested as the underlying gene for a human T1D associated region on chromosome 21. To further evaluate the role of UBASH3A in T1D, we targeted *Ubash3a* in NOD mice using zinc-finger nuclease mediated mutagenesis. In both 10-week-old females and males, significantly more advanced insulitis was observed in UBASH3A-deficient than in wild-type NOD mice. Consistently, UBASH3A-deficient NOD mice developed accelerated T1D in both sexes, which was associated with increased accumulation of β-cell autoreactive T cells in the spleen and pancreatic lymph node. Adoptive transfer of splenic T cells into NOD.*Rag1*^*-/-*^ mice demonstrated that UBASH3A deficiency in T cells was sufficient to promote T1D development. Our results provide strong evidence to further support a role of UBASH3A in T1D. In addition to T1D, UBASH3A deficiency also promoted salivary gland inflammation in females, demonstrating its broad impact on autoimmunity.

## Introduction

Genetic susceptibility and its interaction with incompletely defined environmental factors promote the development of type 1 diabetes (T1D)^1^. Human genome wide association studies (GWAS) have identified more than 50 genetic loci significantly linked to T1D^2,3^. While the greatest genetic contribution is provided by certain human leukocyte antigen (HLA) haplotypes, non-HLA susceptibility genes also convey significant risk for T1D development. However, our knowledge of the underlying genes within these mapped GWAS regions is incomplete. One such region is on chromosome 21, of which *UBASH3A* has been indicated as the underlying gene. Recent fine mapping studies identified several T1D-associated non-coding single nucleotide polymorphisms (SNPs) in *UBASH3A*^3^. Subsequent studies further linked *UBASH3A* risk alleles to its elevated expression and reduced interleukin (IL)-2 production in human CD4 T cells, providing additional evidence to support it as a causal gene in this T1D region^4,5^.

UBASH3A belongs to the ubiquitin-associated and Src-homology 3 domain containing (UBASH3) family that also includes a second member UBASH3B^6^. Expression of UBASH3A is restricted to lymphoid tissues and primarily in T cells^7^. On the other hand, UBASH3B is ubiquitously expressed^8^. An earlier study indicated that T cells deficient in both UBASH3A and UBASH3B were hyperreactive to T cell receptor (TCR) stimulation and the double knockout mice were more susceptible to experimental autoimmune encephalomyelitis compared to the wild-type control^7^. More recently, it was demonstrated that deficiency in either UBASH3A or UBASH3B alone had distinct effects in promoting trinitrobenzene sulfonic acid induced colitis in mice^9^. UBASH3B suppresses TCR signaling by dephosphorylating ZAP-70 and Syk^7,10,11^. On the other hand, UBASH3A has very week phosphatase activity but can suppress T cell activation by diminishing NF-κB signal transduction, downregulating the cell surface TCR-CD3 complex, and inhibiting CD28-mediated costimulation^4,12^.

Genetic manipulation in animal models remains an important approach to provide functional evidence and to conduct mechanistic studies of disease susceptibility genes. NOD mice develop spontaneous autoimmune diabetes and have been used for T1D research for three decades^13,14^. As T1D is a complex disease, the impact of a single gene in autoimmune diabetes is more likely to be observed in the NOD strain that provides the susceptible genetic background. One approach to test the role of a human T1D candidate gene in NOD mice is to target the mouse ortholog and determine if its deficiency affects diabetes progression. Here, we used zinc-finger nucleases (ZFNs) to target *Ubash3a* in the NOD strain to further evaluate its role in T1D.

## Materials and methods

### Mice

**Figure 1:**
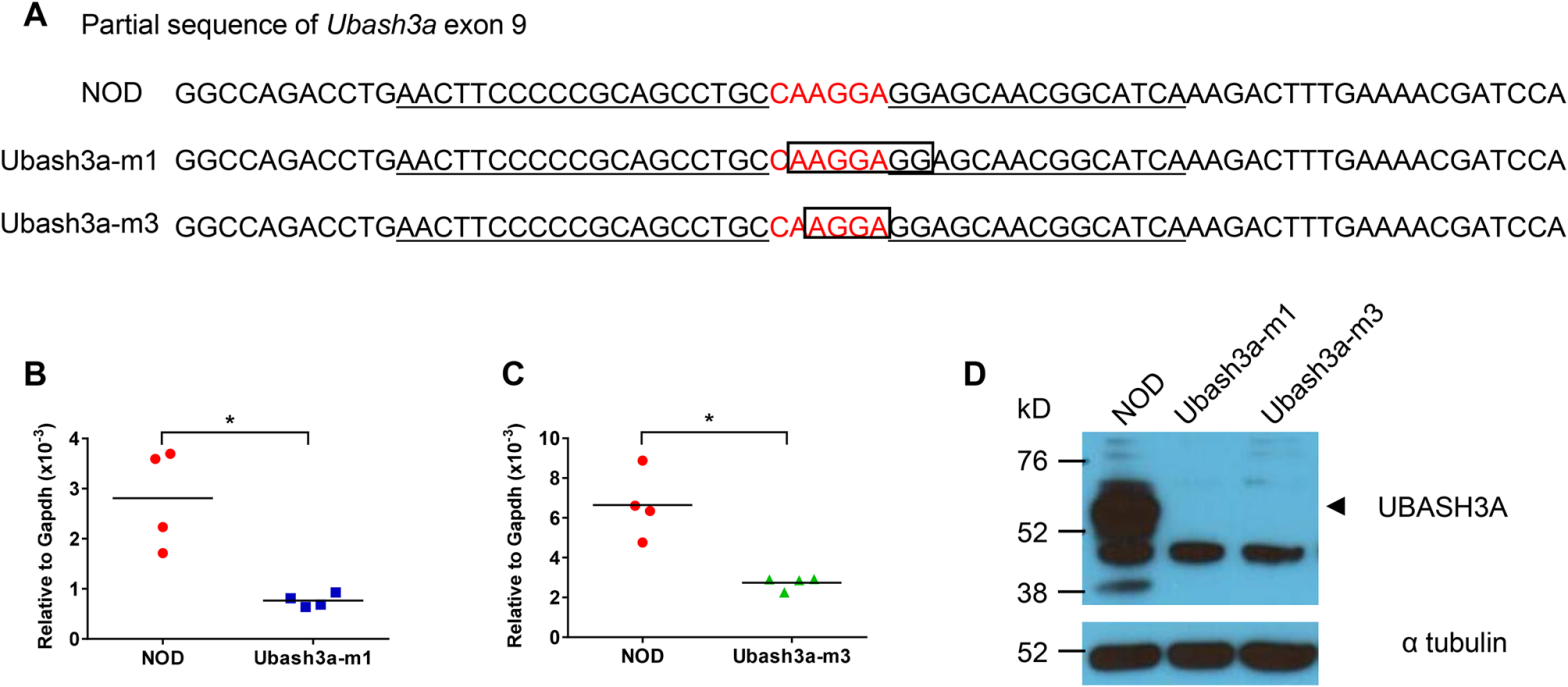
Generation of *Ubash3a* knockout NOD mice. **A** Zinc-finger nuclease (ZFN)-mediated mutagenesis of the *Ubash3a* gene. The partial exon 9 sequence of the wild-type NOD *Ubash3a* is shown at the top. The deleted nucleotides in *Ubash3a* mutant strains, Ubash3a-m1 and Ubash3a-m3, were determined by DNA sequencing and are indicated by a box and shown below the wild-type sequence. The ZFN target site is shown in red and each of the ZFN binding sequences on the opposite strands is underlined. **B**, **C** Expression of *Ubash3a* is significantly lower in Ubash3a-m1 (**B**) and Ubash3a-m3 (**C**) strains than in wild-type NOD mice. The relative levels of *Ubash3a* expression in total thymocytes between indicated strains were determined by TaqMan gene expression assays. The level of *Ubash3a* expression is normalized to that of *Gapdh*. Each symbol represents one mouse. The horizontal lines indicate the means. *P < 0.05 by Mann–Whitney test. **D** UBASH3A protein expression is not detectable in Ubash3a-m1 or Ubash3a-m3 mice. Western blotting using UBASH3A-specific antibody was performed to analyze its protein expression in the thymus of the indicated strains. The expected size of UBASH3A is indicated by an arrowhead. The result is a representative of two independent experiments. The full-length blots are shown in Supplementary Fig. 1.

NOD/ShiLtJ (NOD) and NOD.129S7(B6)-*Rag1*^*tm1Mom*^/J (NOD.*Rag1*^*−/−*^) mice were originally obtained from The Jackson Laboratory and subsequently maintained at the Medical College of Wisconsin (MCW). Ubash3a-m1 and Ubash3a-m3 strains with targeted mutations in *Ubash3a* were generated by ZFN-mediated mutagenesis. Constructs of the ZFN pairs that specifically target *Ubash3a* were designed, assembled, and validated by Sigma-Aldrich. The ZFN binding and targeting sequences of the *Ubash3a* gene as well as the mutant sequences in Ubash3a-m1 and Ubash3a-m3 strains are shown in Fig. 1A. All procedures used to generate gene targeted mutations in NOD mice using ZFNs have been previously described^15^. Successful targeting was identified by PCR-amplifying genomic DNA using forward (5′-CACAAACGACATCCTTGGC-3′) and reverse (5′-GCAGGGGCTCAGTGGATAC-3′) primers, followed by Sanger sequencing of the PCR products. All mouse experimental protocols were carried out in accordance with the MCW Institutional Animal Care and Use Committee guidelines and approved by the committee.

### T1D incidence study and histological analysis of insulitis

Assessment of diabetes and insulitis were performed as previously described^16^. Mice were tested weekly for glycosuria (Bayer Diastix) and considered diabetic with two consecutive readings > 250 mg/dL. Pancreata from 10-week-old non-diabetic mice were fixed in 10% neutral buffered formalin and 4 µm sections were cut, discarding 60 µm between sections. The pancreatic sections were then stained with aldehyde fuchsin followed by a hematoxylin and eosin (H&E) counterstain. Islets on four sections were scored per mouse and the mean insulitis score was calculated for each mouse. The insulitis scores were determined as follows: 0-no infiltration, 1-leukocytes surrounding islet but no penetration, 2-estimated loss of up to 25% of the β cells, 3-estimated loss of up to 75% of the β cells, 4-end stage, less than 25% of the β cells remaining.

### Flow cytometry analysis

Anti-CD3ε (145-2C11), Anti-CD4 (GK1.5), anti-CD8α (53–6.7), anti-CD44 (IM7), and anti-FOXP3 (FJK-16 s) used for flow cytometry analysis were purchased from eBioscience or BD Biosciences. Antibody staining and flow cytometry analysis procedures have been described previously^17^ with the exception of major histocompatibility complex (MHC) tetramer staining. MHC class I and II tetramers were obtained from the NIH tetramer core facility. MHC class I (K^d^) tetramers were loaded with an islet specific glucose-6-phosphatase catalytic subunit related protein (IGRP) peptide (VYLKTNVFL, amino acid residues 206–214). MHC class II (A^g7^) tetramers were loaded with a BDC2.5 mimotope peptide (AHHPIWARMDA) or a control peptide (PVSKMRMATPLLMQA). For detecting IGRP_206-214_ reactive CD8 T cells, samples were incubated with MHC class I tetramers at room temperature for 15 min, followed by staining with the antibody cocktail (anti-CD4, anti-CD8, and anti-CD44) at 4 °C for 30 min. For detecting autoreactive CD4 T cells, samples were incubated with both BDC2.5 and control MHC class II tetramers in the complete RPMI medium for 1 h at 37 °C, followed by staining with the antibody cocktail (anti-CD4, anti-CD8, and anti-CD44) at 4 °C for 30 min. Dead cells were excluded by 7-AAD staining. Intracellular staining for FOXP3 was performed using the FOXP3 staining buffer set from eBioscience. Stained cells were run on an LSRII flow cytometer (BD Biosciences) and analyzed using FlowJo software (Tree Star).

### In vitro T cell proliferation assay

Splenocytes from NOD and Ubash3a-m3 mice were stained with 2 µM CFSE (Invitrogen) at 37ºC for 10 min and washed four times with complete RPMI. Cells were cultured with 0.1 or 1 µg/mL anti-CD3 (145-2C11, eBioscience) for three days. Cultured cells were then washed with FACS buffer and stained with anti-CD4, anti-CD8, and 7AAD. Flow cytometry was used to measure proliferation of T cells by CFSE dilution.

### Adoptive T-cell transfer

Splenic total T cells were isolated by negative selection using Pan T cell isolation kit II (Miltenyi Biotec). NOD.*Rag1*^*-/-*^ mice were injected *i.v.* with 5 × 10^6^ purified T cells (> 92% pure) to test their diabetogenic activity.

### Reverse transcription and quantitative polymerase chain reaction (qPCR)

Reverse transcription and qPCR were performed as previously described^18^. TaqMan gene expression assays used were Mm99999915_g1 (*Gapdh*) and Mm01348237_m1 (*Ubash3a*), which were purchased from Applied Biosystems.

### Western blotting

Cells were lysed in RIPA buffer (Boston Bioproducts) supplemented with SigmaFast protease inhibitors (Sigma Aldrich). Protein samples (20 µg) were separated on 10% SDS–polyacrylamide gels and then transferred to a polyvinylidene difluoride membrane. Immunoblotting was performed using rabbit polyclonal antibodies directed against mouse UBASH3A at 4 ºC overnight, followed by goat anti-rabbit secondary antibody (Millipore, 12–348) at room temperature for 45 min. The rabbit anti-UBASH3A polyclonal antibody was produced by Thermo Scientific using the oligopeptide EKLQEFWRESRRQCAKNR (amino acid residues 86–103) as an antigen. Binding of the secondary antibody was detected by the Western bright ECL detection kit (Advansta Corporation). The membrane was then stripped with Restore plus Western Blot stripping buffer (Thermo Scientific) and re-probed with the rabbit anti-α tubulin polyclonal antibody (Abcam, ab4074).

### Histology of lacrimal and salivary glands

Submandibular salivary glands and exorbital lacrimal glands were fixed in buffered formalin, processed, and embedded in paraffin. One five µm section of paired lacrimal glands and paired salivary glands from each mouse was stained with H&E, and inflammation was quantified using standard focus scoring^19^. Briefly, slides were analyzed by light microscopy at 10 × magnification to determine the number of mononuclear cell foci in tissue sections of salivary or lacrimal glands, with a focus defined as an aggregate of at least 50 mononuclear cells. Digital images were obtained by scanning slides with a PathScan Enabler IV (Meyer Instruments, Houston, TX), and tissue areas were measured using ImageJ software (US National Institutes of Health, Bethesda, MD, USA)^20^. Focus scores were calculated as number of foci per 4 mm^2^ tissue area. Analyses were performed in a blinded manner. The wild-type male lacrimal and female salivary gland focus scores represent a subset of our recently published larger wild-type data set^21^ that were chosen based on being colony-matched and temporally-matched (i.e., procured within a year of procurement of the knockout samples to which they are compared). Higher magnification representative images were captured with a 10X objective on a Leitz DM-RB research microscope with a Leica DCF700T digital camera using the Leica Application Suite X software (Leica Microsystems, Wetzlar, Germany).

## Results

### Generation of UBASH3A-deficient NOD mice

To further study the role of UBASH3A in T1D, we established two different lines of *Ubash3a* mutant strains with 7 and 4 base-pair deletions in exon 9 (Ubash3a-m1 and Ubash3a-m3 respectively, Fig. 1A). Quantitative RT-PCR demonstrated that the expression of *Ubash3a* in the thymus was significantly reduced in mutant mice compared to the wild-type control, suggesting a result of nonsense-mediated decay (Fig. 1B, C). Western blotting confirmed the absence of UBASH3A protein expression in the thymi of Ubash3a-m1 and Ubash3a-m3 mice (Fig. 1D and Supplementary Fig. 1). The predicted full-length UBASH3A is indicated by an arrowhead. The lower band that appeared in the wild-type (~ 38kD) but not mutants may result from alternative splicing as reported in human *UBASH3A* ^5^.

### Accelerated insulitis and T1D development in UBASH3A-deficient NOD mice

**Figure 2:**
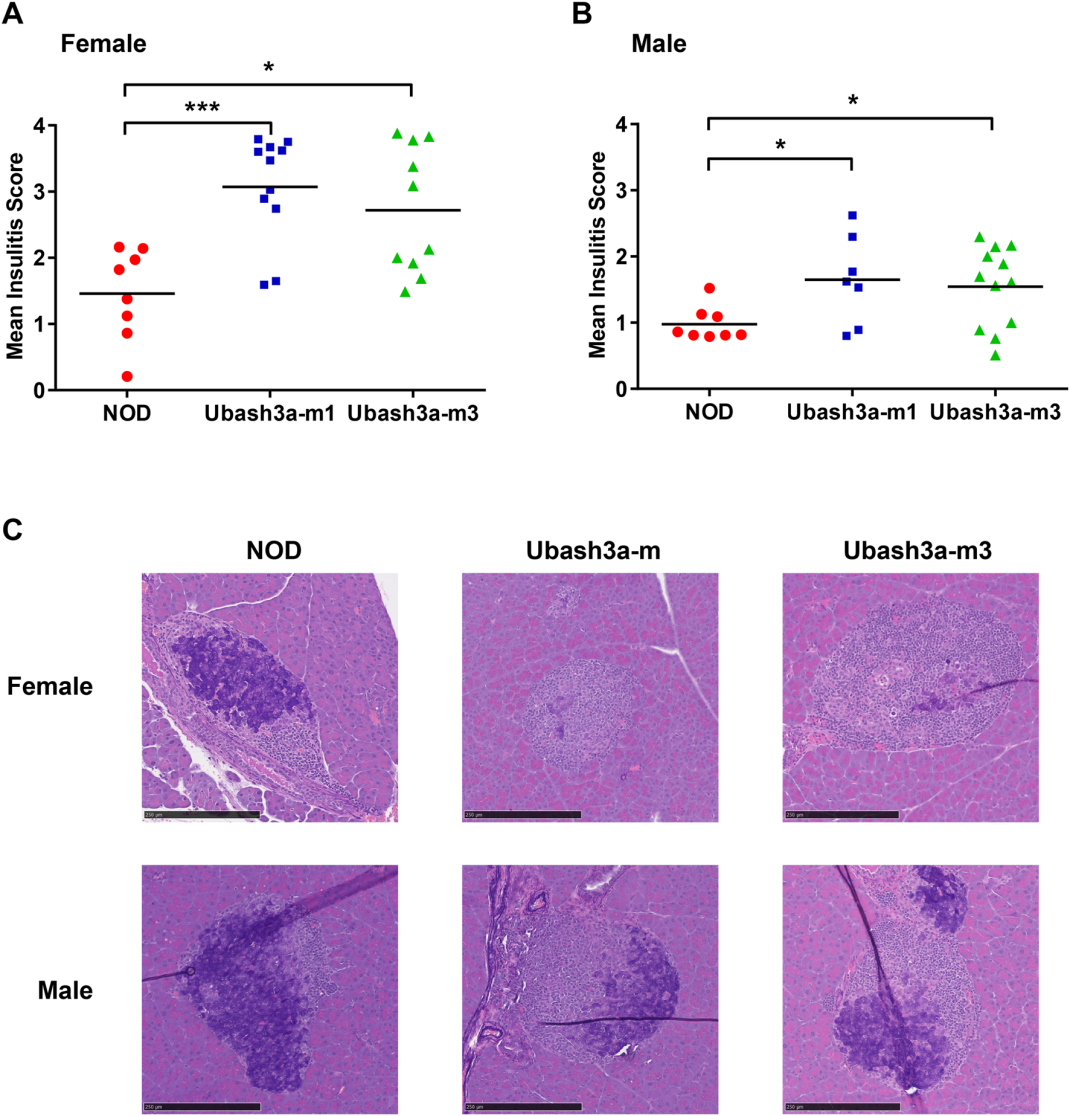
UBASH3A deficiency promotes insulitis in NOD mice. **A**, **B** Mean insulitis scores were determined in pre-diabetic 10-week-old female (**A**) and male (**B**) mice of the indicated strains. Pancreatic islets were individually scored as follows: 0, no lesions; 1, peri-insulitis but no penetration; 2, up to 25% islet destruction; 3, up to 75% islet destruction; and 4, end stage to complete islet destruction (see Supplementary Fig. 2 for representative islets of each score). Four pancreatic sections were examined for each mouse. At least 20 islets were scored for each mouse and the mean was calculated, except three Ubash3a-m1 and three Ubash3a-m3 females where less than 20 islets were available for the analysis. The number of islets analyzed in each mouse and the distribution of scores are provided in Supplementary Table 1. Each symbol represents one mouse (female: n = 8 for wild-type, n = 11 for Ubash3a-m1, n = 10 for Ubash3a-m3; male: n = 8 for wild-type, n = 7 for Ubash3a-m1, n = 12 for Ubash3a-m3). The horizontal lines indicate the means. Mean insulitis scores are significantly higher in both *Ubash3a* mutant lines than in wild-type NOD mice (*P < 0.05, ***P < 0.005 by Mann–Whitney test). **C** Representative images of aldehyde fuchsin and H&E stained islets from each strain. The scale bar is 250 µm.

**Figure 3:**
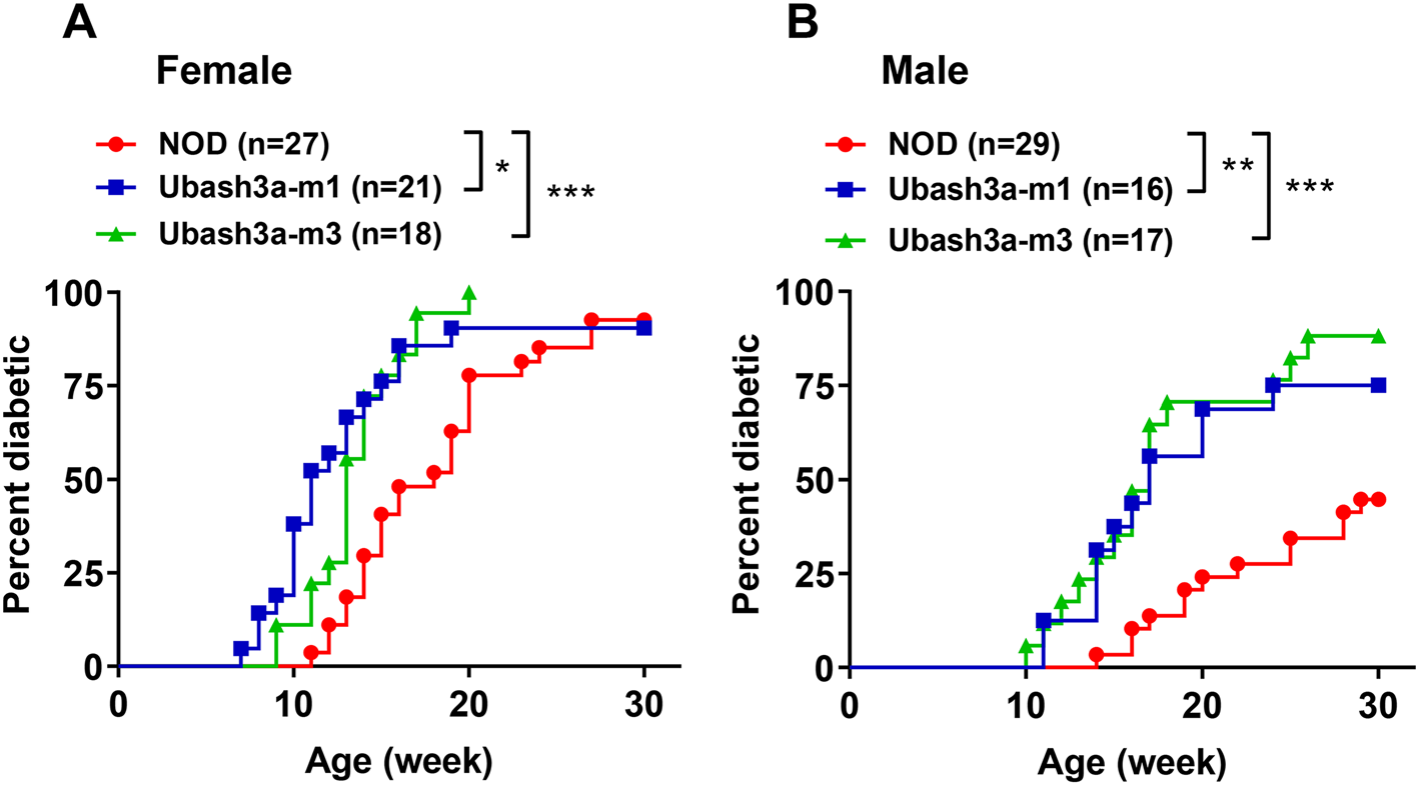
T1D is accelerated in UBASH3A-deficient NOD mice. **A** Female and **B** male mice of the indicated strains were monitored weekly for T1D development for 30 weeks. Diabetes onset was determined by two consecutive positive readings of glycosuria on a urine test strip (> 250 mg/dL). T1D progression in Ubash3a-m1 and Ubash3a-m3 strains was significantly accelerated in both sexes when compared to wild-type NOD mice (*P < 0.05; **P < 0.01; ***P < 0.005 by Log-rank test).

We first analyzed insulitis at 10 weeks of age by histology. Compared to wild-type NOD mice, the level of islet infiltration was increased in Ubash3a-m1 and Ubash3a-m3 strains resulting in significantly higher mean insulitis scores in both females and males (Fig. 2 and Supplementary Table 1). In line with the results of histological analysis, T1D incidence studies showed significant acceleration in both sexes of Ubash3a-m1 and Ubash3a-m3 strains compared to wild-type NOD mice (Fig. 3). Some UBASH3A-deficient female mice developed T1D prior to 10 weeks of age, which was rarely observed in our NOD colony. As Ubash3a-m1 and Ubash3a-m3 strains had similar T1D incidence, we focused on the Ubash3a-m3 stock for additional phenotypic analyses.

### UBASH3A deficiency in T cells promotes their diabetogenic activity

**Figure 4:**
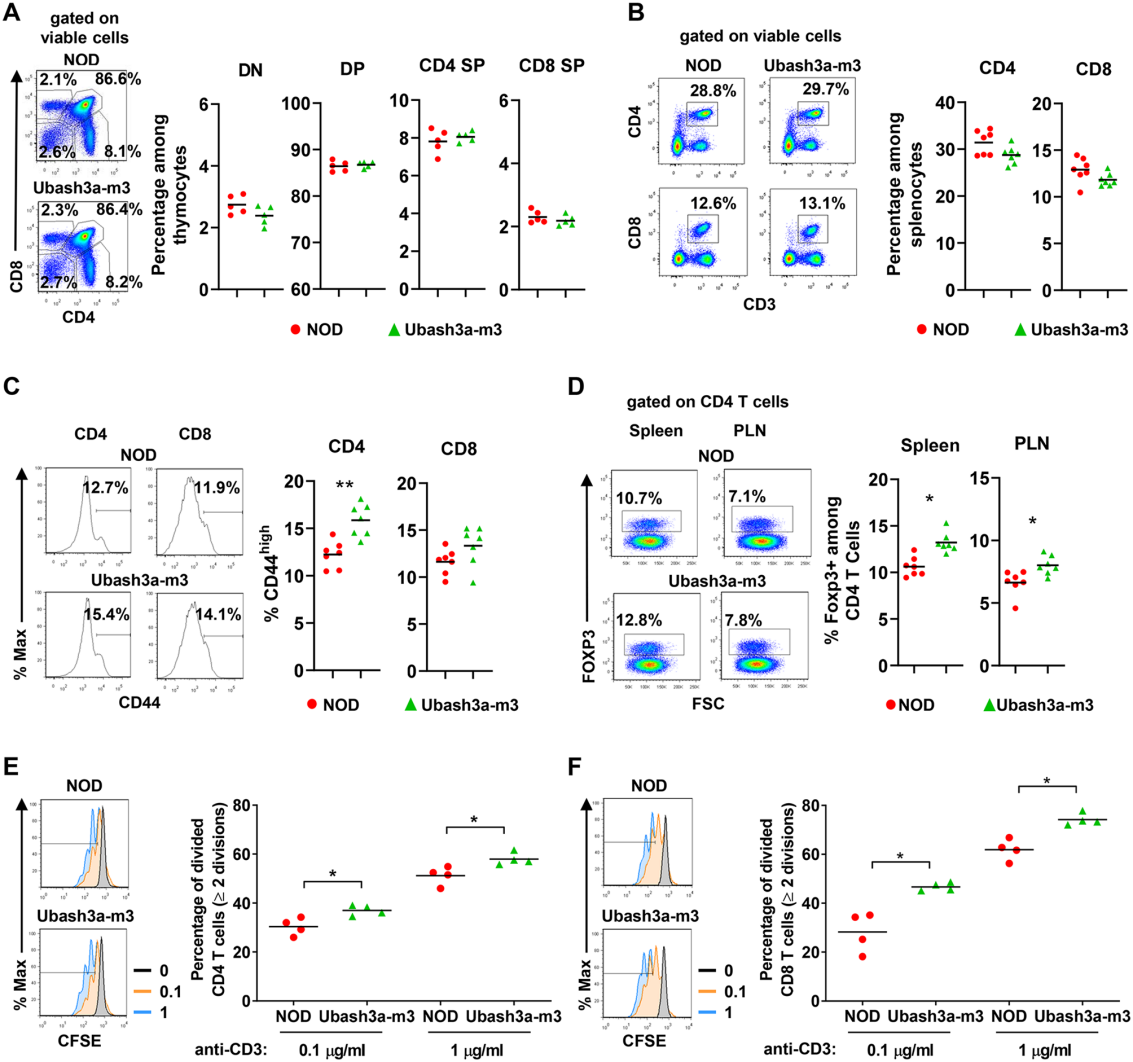
The impact of UBASH3A deficiency on T cell populations. **A** The frequencies of double negative (DN), double positive (DP), CD4 single positive (SP), and CD8 SP thymocytes in 7-week-old NOD and Ubash3a-m3 males (n = 5 per strain). **B** The frequencies of splenic CD4 and CD8 T cells in 7-week-old NOD and Ubash3a-m3 males (n = 7 per strain). **C** The frequencies of CD44^high^ cells among splenic CD4 and CD8 T cells in in 7-week-old NOD and Ubash3a-m3 males (n = 7 per strain). **D** The frequencies of FOXP3^+^ Tregs among CD4 T cells in the spleens and PLNs of 7–9-week-old NOD and Ubash3a-m3 males (n = 7 per strain). **E**, **F** In vitro proliferation of NOD and Ubash3a-m3 CD4 (**E**) and CD8 (**F**) T cells. Splenocytes from 11-week-old NOD and Ubash3a-m3 males were labeled with CFSE and stimulated with indicated concentrations of anti-CD3 in culture for 3 days. The frequency of cells that have divided twice or more among viable CD4 or CD8 T cells was determined by flow cytometry. The representative flow cytometry plots are shown on the left and the summarized results are shown on the right. In all summary plots, each symbol represents one mouse. The horizontal lines indicate the means. *P < 0.05, **P < 0.01 by Mann–Whitney test.

UBASH3A is primarily expressed in T cells^7^. Thus, we determined if UBASH3A deficiency affects T cell populations. The number of thymocytes was similar in 7-week-old NOD and Ubash3a-m3 mice (3.88 ± 0.28 × 10^7^ versus 4.58 ± 0.42 × 10^7^, respectively, n = 10). The percentages of double negative (DN), double positive (DP), CD4 single positive (SP), and CD8 SP thymocytes were comparable between NOD and Ubash3a-m3 mice (Fig. 4A). The overall cell number in the spleen was also similar in 7-week-old NOD and Ubash3a-m3 mice (5.90 ± 0.22 × 10^7^ versus 7.15 ± 0.46 × 10^7^, respectively, n = 7). The frequencies of CD4 and CD8 T cells in the spleen were also comparable in NOD and Ubash3a-m3 mice (Fig. 4B). The proportions of CD44^high^ cells among CD4 and CD8 T cells in the spleen were marginally higher in Ubash3a-m3 than in NOD mice and reached statistical significance in CD4 T cells (Fig. 4C). We also analyzed the percentage of CD4^+^ FOXP3^+^ regulatory T cells (Tregs) and found their frequencies slightly but significantly increased in the spleens and pancreatic lymph nodes (PLNs) of 7–9-week-old Ubash3a-m3 mice compared to the age-matched NOD control (Fig. 4D and Supplementary Fig. 3A). This may result from a counteracting response to heightened T cell activation in UBASH3A-deficient mice as no difference was observed in the thymus (data not shown). To initially test if the function of T cells is affected by UBASH3A deficiency, we analyzed their proliferation in vitro upon anti-CD3 stimulation. Consistent with its role in controlling TCR signaling, UBASH3A-deficient CD4 and CD8 T cells proliferated more extensively than the wild-type counterparts (Figs. 4E, F).

**Figure 5:**
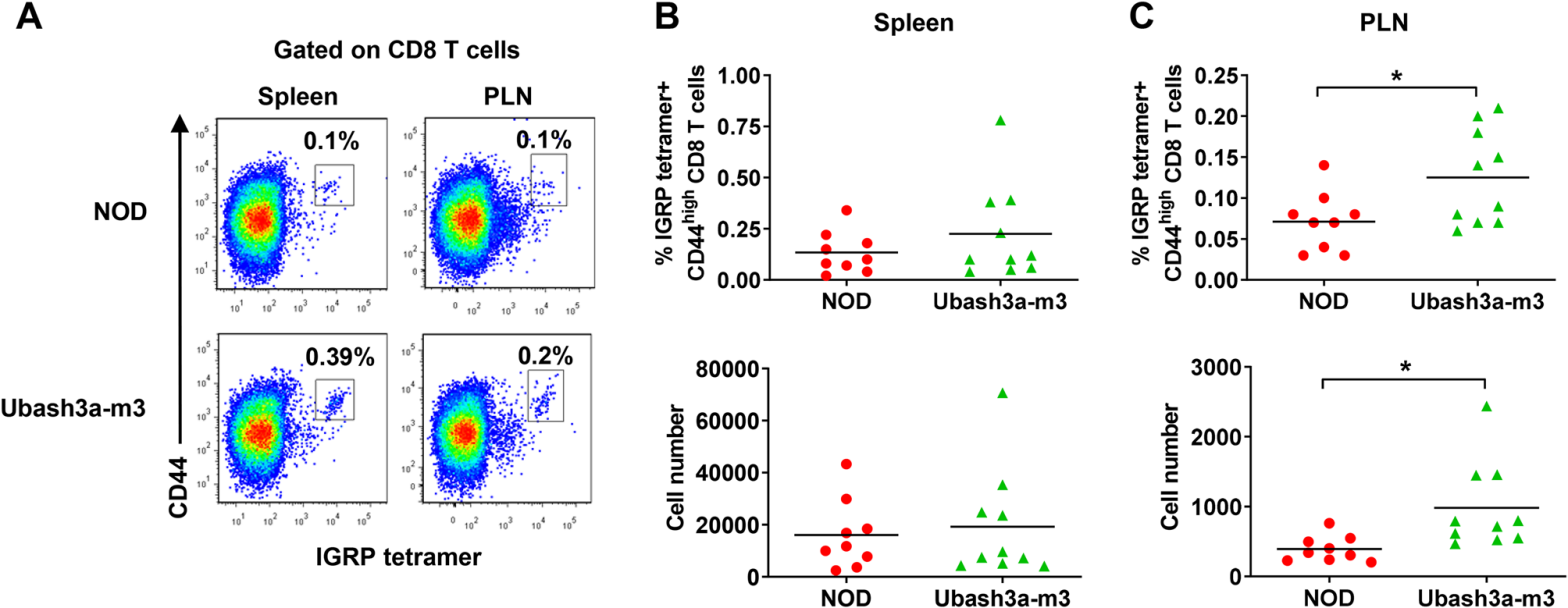
*Ubash3a* mutant mice have increased accumulation of β-cell autoreactive CD8 T cells compared to wild-type NOD mice. **A** Representative flow cytometry plots of MHC class I tetramer staining. **B**, **C** The percentage and number of IGRP_206-214_ reactive CD8 T cells were analyzed by MHC class I tetramers in the spleens (**B**) and PLNs (**C**) of 9–10-week-old wild-type NOD (n = 9) and Ubash3a-m3 (n = 10) female mice. Cells were stained with anti-CD4, anti-CD8, anti-CD44, and IGRP_206-214_ loaded MHC class I tetramers. The percentages and numbers of CD44^high^ tetramer-positive cells among viable CD4^-^ CD8^+^ were determined. Each symbol represents one mouse. The horizontal lines indicate the means. *P < 0.05 by Mann–Whitney test.

**Figure 6:**
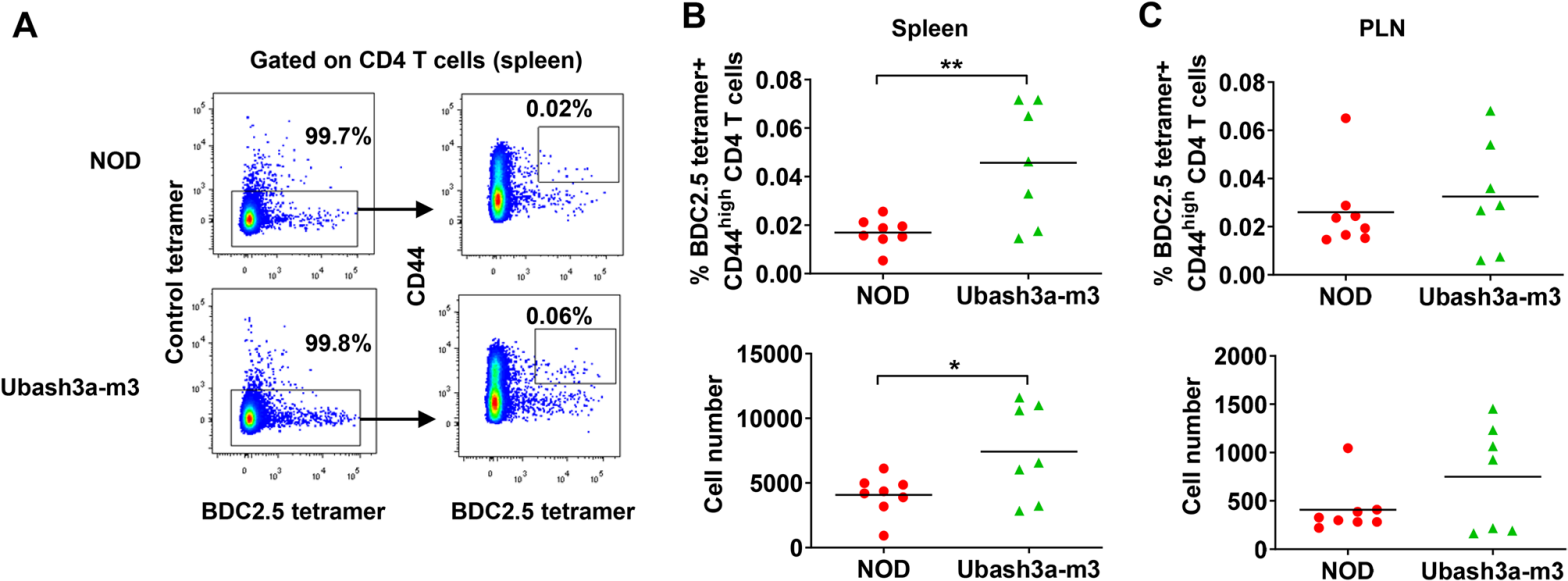
*Ubash3a* mutant mice have increased accumulation of β-cell autoreactive CD4 T cells compared to wild-type NOD mice. **A** Representative flow cytometry plots of MHC class II tetramer staining. **B**, **C** The percentage and number of BDC2.5-like CD4 T cells were analyzed by MHC class II tetramers in the spleens (**B**) and PLNs (**C**) of 10–11-week-old wild-type NOD (n = 8) and Ubash3a-m3 (n = 7) female mice. Cells were stained with anti-CD4, anti-CD8, anti-CD44, as well as negative control or BDC2.5 mimotope peptide loaded MHC class II tetramers. The percentages and numbers of CD44^high^ control tetramer-negative and BDC2.5 tetramer-positive cells among viable CD4^+^ CD8^-^ were determined. Each symbol represents one mouse. The horizontal lines indicate the means. *P < 0.05 by Mann–Whitney test.

Next, we asked if UBASH3A controls the accumulation of β-cell autoreactive T cells in NOD mice. We used MHC class I tetramers to identify IGRP_206-214_ reactive CD8 T cells in the PLNs and spleens of 9–10-week-old NOD and Ubash3a-m3 females. CD44 expression was analyzed to identify activated T cells. The Ubash3a-m3 strain had significantly higher frequencies and numbers of activated CD44^high^ IGRP_206-214_ reactive CD8 T cells in the PLN, albeit not in the spleen (Fig. 5 and Supplementary Fig. 3B). We also used MHC class II tetramers loaded with a mimotope peptide recognized by BDC2.5 diabetogenic CD4 T cells. Compared to NOD mice, the Ubash3a-m3 strain had significantly increased frequencies and numbers of activated CD44^high^ BDC2.5-like cells in the spleen, but not in the PLN (Fig. 6 and Supplementary Fig. 3C). These results suggested that UBASH3A deficiency perturbed the pathogenic T cell compartment and led to enhanced accumulation of β-cell autoreactive T cells in NOD mice.

**Figure 7:**
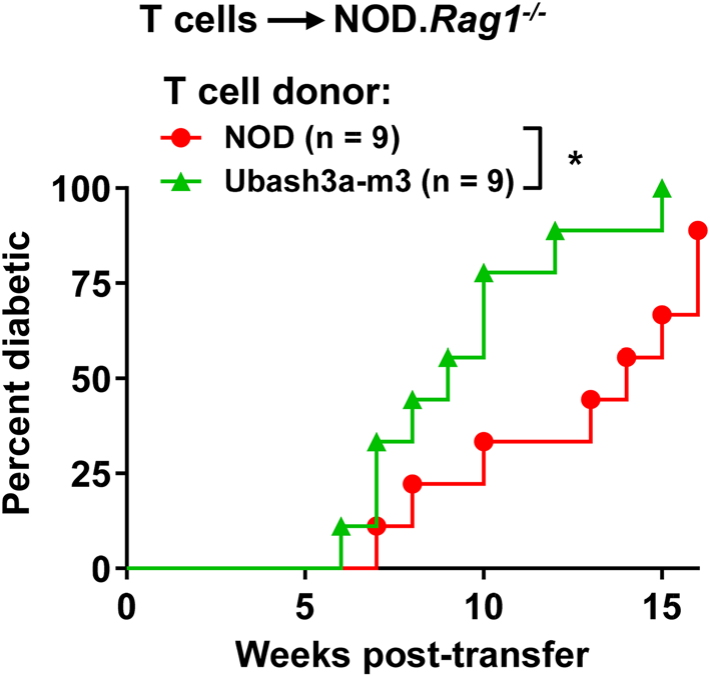
UBASH3A-deficient T cells are more diabetogenic than the wild-type counterparts. Purified splenic T cells (5 × 10^6^) isolated from 6 to 8-week-old wild-type NOD or Ubash3a-m3 females were transferred into 6 to 8-week-old sex-matched NOD.*Rag1*^*-/-*^ recipients. A similar 2:1 ratio of CD4 and CD8 T cells was present in the purified total wild-type and Ubash3a-m3 T cells. T1D development was monitored weekly for 16 weeks. Diabetes onset was determined by two consecutive positive readings of glycosuria on a urine test strip (> 250 mg/dL). T1D incidence of Ubash3a-m3 T cell recipients is significantly different from those infused with wild-type T cells (*P < 0.05 by Log-rank test).

We next asked if UBASH3A deficiency in T cells is sufficient to alter their diabetogenic activity. To test this, we adoptively transferred purified splenic T cells from 6 to 8-week-old NOD or Ubash3a-m3 females into sex-matched NOD.*Rag1*^*-/-*^ mice. NOD.*Rag1*^*-/-*^ mice do not have mature T and B cells. As a result, they do not develop spontaneous T1D and can be used as recipients to test the diabetogenic activity of T cells. T cells isolated from the Ubash3a-m3 strain induced significantly more rapid onset of T1D in the NOD.*Rag1*^*-/-*^ recipients compared to those isolated from the wild-type mice (Fig. 7). Collectively, these results indicate that UBASH3A deficiency leads to elevated β-cell destruction and rapid onset of T1D in part through promoting the diabetogenic activity of T cells.

### UBASH3A regulates female salivary gland inflammation

**Figure 8:**
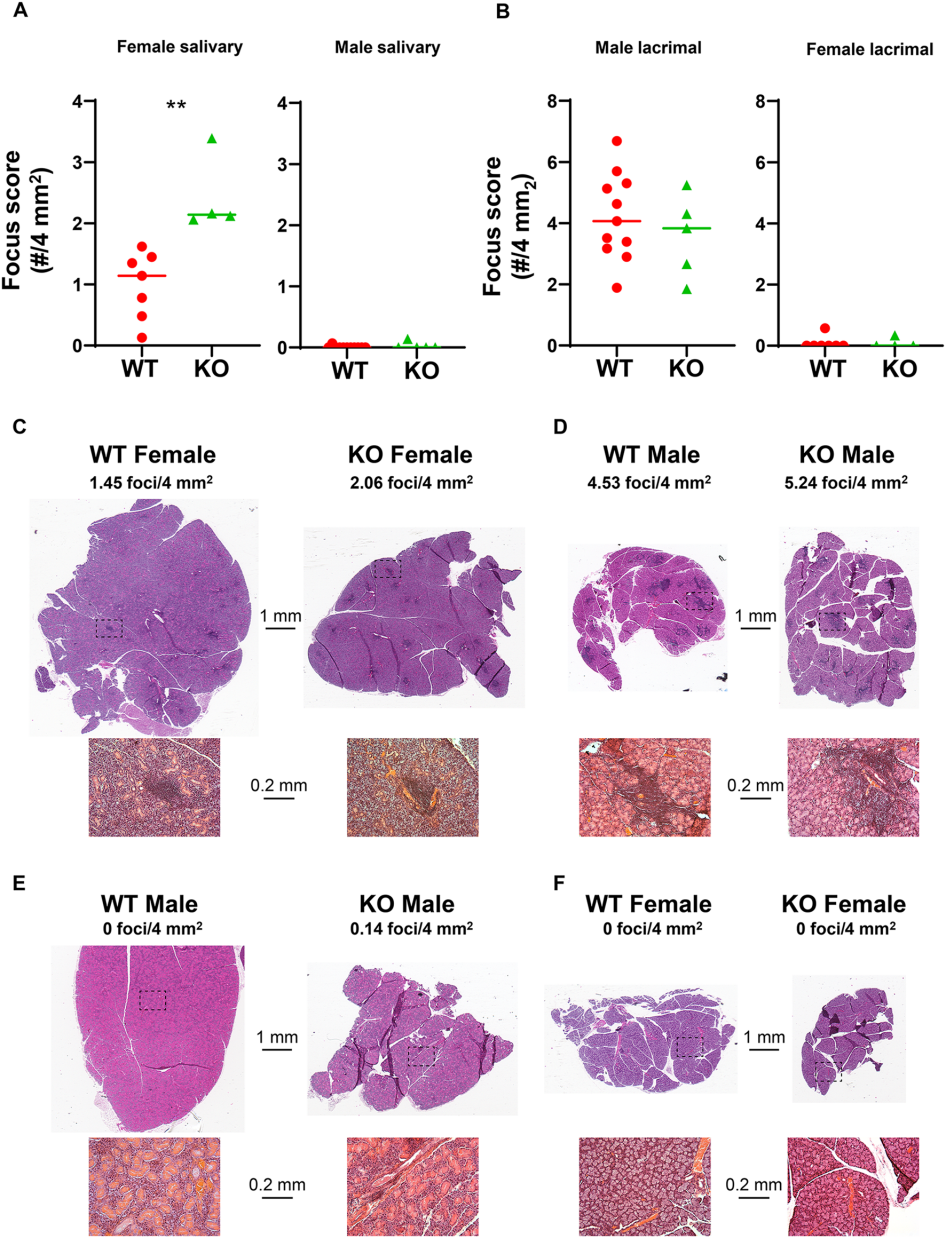
UBASH3A controls female salivary gland inflammation in NOD mice. **A**, **B** Quantification of inflammation of salivary glands (**A**) and lacrimal glands (**B**) from 10-week-old pre-diabetic wild-type NOD (WT) and Ubash3a-m3 (KO) mice using standard focus scoring. One five μm section of paired lacrimal glands and paired salivary glands from each mouse was stained with H&E. A focus was defined as a cluster of at least 50 mononuclear cells, and focus scores were calculated as number of foci per 4 mm^2^ tissue area. Female and male glands are presented in separate graphs as indicated. Symbols represent individual mice including female WT (n = 7), female KO (n = 4), male WT (n = 11), and male KO (n = 5). Lines are medians. **P < 0.001 by Mann–Whitney test. **C**–**E** Histology images show representative H&E-stained sections of female salivary (**C**), male lacrimal (**D**), male salivary (**E**) and female lacrimal (**F**) glands of WT or KO mice as indicated. Dashed boxes represent area shown in higher-magnification image below each representative image. Scale bars for low or high magnification images are indicated between set of images.

In addition to T1D, NOD mice spontaneously develop autoimmunity of salivary and lacrimal glands, and are a well-established model of Sjögren syndrome^22^. To determine if disruption of *Ubash3a* affected Sjögren syndrome-like manifestations that spontaneously develop in NOD mice, we quantified inflammation in salivary and lacrimal glands of 10-week-old pre-diabetic wild-type NOD and Ubash3a-m3 mice. In NOD mice, Sjögren syndrome-like manifestations occur in a sex-specific manner with females spontaneously developing salivary gland inflammation and males spontaneously developing lacrimal gland inflammation^23,24,25,26,27,28^. In female salivary glands, Ubash3a-m3 mice displayed a higher degree of inflammation compared to age-matched wild-type NOD mice (Fig. 8A, C). In contrast, male lacrimal gland inflammation was not different between Ubash3a-m3 and wild-type NOD mice (Fig. 8B, D), and no inflammation developed in the normally unaffected organs, i.e., male salivary or female lacrimal glands (Fig. 8A, B, E, F). Thus, UBASH3A deficiency preferentially enhanced female salivary gland inflammation but did not alter lacrimal gland autoimmunity.

## Discussion

In this report, we sought to determine if UBASH3A controls diabetes development in NOD mice to support its role in human T1D. We describe successful generation of new NOD strains with targeted mutations in the murine ortholog of the human T1D candidate gene *UBASH3A*. We demonstrated that UBASH3A deficiency accelerated insulitis and T1D progression in both sexes and provided evidence to support that UBASH3A regulates autoimmune diabetes through its expression in T cells. UBASH3A deficiency also led to enhanced salivary gland infiltration in female NOD mice. Thus, UBASH3A plays a broader role in regulating autoimmunity beyond T1D, consistent with its association with other autoimmune diseases in humans^29,30,31^.

UBASH3A is structurally similar to UBASH3B, both of which negatively regulate TCR signaling and T cell activation^4,7,10,11,12,32,33,34^. The ability of UBASH3B to suppress TCR signaling is largely attributed to its phosphatase activity that dephosphorylates ZAP-70 and Syk^7,10,11^. However, UBASH3A has very limited phosphatase activity^11,35,36^. Thus, other mechanisms are likely employed by UBASH3A to inhibit the TCR signaling pathway. Indeed, recent studies in human Jurkat cells have revealed that UBASH3A can modulate T cell activation through multiple mechanisms^4,12^. UBASH3A diminishes NF-κB signal transduction downstream of TCR stimulation by suppressing the activation of the IKK complex, possibly through its interaction with TAK1 and NEMO^4^. In addition, UBASH3A can downregulate the cell surface TCR-CD3 complex likely through its interaction with proteins involved in the endoplasmic reticulum associated protein degradation pathway, components of 26S proteasome, and components of the endocytic recycling pathway^12^. UBASH3A also directly binds to the E3 ubiquitin ligase Cbl-b that inhibits CD28-mediated costimulation for T cell activation^12^. It remains to be determined if these diverse mechanisms shown in human Jurkat cells are also utilized by UBASH3A to attenuate activation of murine T cells. Supporting this possibility is the recent finding that UBASH3A is part of the Cbl-b signalosome in primary murine CD4 T cells^37^. Additionally, UBASH3A may regulate β-cell autoreactive T cells independent of TCR and costimulation pathways as it has been shown to increase T cell death through binding to apoptosis-inducing factor (AIF), a mitochondrial protein that triggers caspase-independent cell death under stress conditions^38^.

Several non-coding SNPs in *UBASH3A* have been associated with human T1D^5^. Studies linking genotypes to the level of *UBASH3A* transcript indicate that the risk alleles enhance its expression^4,5^. In addition, the *UBASH3A* risk variants are associated with reduced IL-2 production in primary human CD4 T cells upon stimulation, which is hypothesized to contribute to dysregulated balance between regulatory and effector T cells in T1D^4,5^. NOD T cells are known to produce less IL-2 compared to those from T1D resistant strains due to polymorphisms in the *Il2* gene within the *Idd3* susceptibility region^39^. The amount of IL-2 production directly correlated with the frequency and function of FOXP3^+^ Tregs in *Idd3* congenic NOD mice^39^. It is likely that increased activation of UBASH3A-deficient T cells also leads to enhanced IL-2 production. Consistent with this idea, we observed that heightened T cell activation was accompanied with increased FOXP3^+^ Tregs in UBASH3A-deficient NOD mice. However, it is apparent that the small increase of FOXP3^+^ Tregs is insufficient to suppress pathogenic T cells and T1D progression. In contrast to the link between the risk alleles and increased *UBASH3A* expression in humans, our mouse study indicates that UBASH3A deficiency leads to accelerated T1D progression. It may appear that the *Ubash3a* knockout allele in NOD mice does not mimic the risk variants in human *UBASH3A*. While it is currently difficult to reconcile the mouse and human genetic studies, several speculative possibilities could explain the discrepancy. First, previous analysis of *UBASH3A* expression was performed in total CD4 T cells. It is not known if various CD4 T cell subsets express different levels of *UBASH3A*. The enhanced *UBASH3A* expression in subjects carrying the risk alleles may reflect a proportional change of different CD4 T cell subsets. It is also not known if *UBASH3A* expression is differentially regulated in CD8 T cells. Second, the *UBASH3A* risk variants may initially reduce its expression in T cells but later enhance its level as a compensatory response of increased TCR signaling over time. In the study of PTPN2, it was shown that loss-of-function initially caused primary human CD4 T cells to be hyperreactive to IL-2 stimulation but eventually T cells became hyporeactive to this cytokine possibly due to a compensatory induction of *SOCS3* expression^40^. Lastly, UBASH3A expression within an optimal range may be critical to regulate the T cell compartment. Genetic manipulation that increases *Ubash3a* expression in NOD mice may disturb the T cell compartment in a way that also leads to accelerated T1D development, a possibility that remains to be determined.

Beyond T1D, disruption of *Ubash3a* in NOD mice increased the degree of salivary gland inflammation in females demonstrating the relevance of *Ubash3a* in autoimmunity beyond T1D and the first evidence of a possible role in Sjögren syndrome. Of note, despite the similar T1D-promoting effects of disruption of *Ubash3a* in male and female NOD mice, the effects on Sjögren syndrome-like manifestations only enhanced female-specific salivary gland inflammation but did not alter male-specific lacrimal gland inflammation. This may reflect more distinct immunological pathways driving sex-specific Sjögren syndrome-like disease manifestations in NOD mice as compared to T1D. While T1D development in NOD mice is greater in females, males are not fully protected from disease development. In contrast, the inflammation of lacrimal and salivary glands tends to occur with more absolute sex-specificity. Moreover, evidence supports different interferon (IFN)-mediated immune pathways required for lacrimal and salivary gland disease with male-specific lacrimal gland inflammation dependent on type I IFN signaling^41^ and female-specific salivary gland inflammation dependent on type II IFN signaling^42^. In T1D, the roles of IFN signaling are more complex, but neither type I nor type II IFN signaling is absolutely required for T1D development in either sex^43^. Given these differences, the role of *Ubash3a* in salivary gland disease may be more similarly linked to its role in T1D, whereas perhaps it plays a redundant role in the immune pathways that dominate lacrimal gland autoimmunity. Another difference in lacrimal and salivary gland disease in NOD mice is the role of CD8 T cells. While activated, inflammatory cytokine-producing CD8 T cells were detected in infiltrates of both lacrimal and salivary glands, CD8 T cells were capable of independently transferring lacrimal (but not salivary) gland disease in an adoptive transfer model^19^. Whether *Ubash3a* deficiency enhances salivary gland-specific CD8 T cell pathogenicity or promotes pathogenic CD4 T cells to increase salivary gland inflammation in females is not known, and the lack of salivary or lacrimal gland antigen-specific T cell epitopes precludes the quantitation of these disease-relevant T cells with tetramers. Of note, disruption of *Ubash3a* was not sufficient to drive autoimmunity of the normally unaffected organs. Thus, no females developed lacrimal gland inflammation, and no males developed salivary gland inflammation. We have shown previously that the sex-specific protection of the unaffected organs requires Tregs^44,45^. Together, these studies suggest that *Ubash3a* is not required for Treg-mediated prevention of spontaneous male salivary gland disease or female lacrimal gland disease.

In summary, we generated UBASH3A-deficient NOD mice and characterized them for the development of T1D and Sjögren syndrome-like manifestations. Disruption of *Ubash3a* in NOD mice did not induce normally absent male salivary or female lacrimal gland inflammation but further exacerbated spontaneous female salivary infiltration and T1D progression in both sexes. The data presented here support an immunomodulatory role for UBASH3A that fine-tunes T cell-mediated autoimmunity.

## Supplementary information

Supplementary Legends

Supplementary Figures

Supplementary Table 1
