# Supplementary material for: UBASH3A deficiency accelerates type 1 diabetes development and enhances salivary gland inflammation in NOD mice
Source: Sci Rep. 2020 Jul 21;10:12019. doi: 10.1038/s41598-020-68956-6 (PMC7374577; doi:10.1038/s41598-020-68956-6)
Supplement: Supplementary file 1 — Supplementary Legends [file 41598_2020_68956_MOESM1_ESM.docx]

**Supplementary materials**

**Supplementary Table 1. Score distribution of insulitis and total number of islets analyzed in each mouse.**

**Supplementary Figure 1. Scanned films of western blotting analysis for UBASH3A and α-tubulin.** The areas indicated by the dotted lines are shown in Figure 1.

**Supplementary Figure 2. Representative islet images of scores 0 to 4.** The level of insulitis (leukocyte infiltration) is defined as the following, 0: no lesions (A); 1: peri-insulitis but no penetration (B); 2: up to 25% islet destruction (C); 3: up to 75% islet destruction (D); and 4: end stage to complete islet destruction (E). In panels B-D, regions of leukocyte infiltration are indicated by arrows. The scale bar is 250 µm.

**Supplementary Figure 3**. The flow cytometry gating strategy for FOXP3^+^ Tregs (A), IGRP_206-214_ tetramer staining (B), and BDC2.5 tetramer staining (C).
