# Supplementary material for: UBASH3A deficiency accelerates type 1 diabetes development and enhances salivary gland inflammation in NOD mice
Source: Sci Rep. 2020 Jul 21;10:12019. doi: 10.1038/s41598-020-68956-6 (PMC7374577; doi:10.1038/s41598-020-68956-6)

UBASH3A deficiency accelerates type 1 diabetes development and enhances salivary gland inflammation in NOD mice

Yi-Guang Chen, Ashley E. Ciecko, Shamim Khaja, Michael Grzybowksi, Aron M. Geurts, and Scott M. Lieberman

Supplementary Figure 1

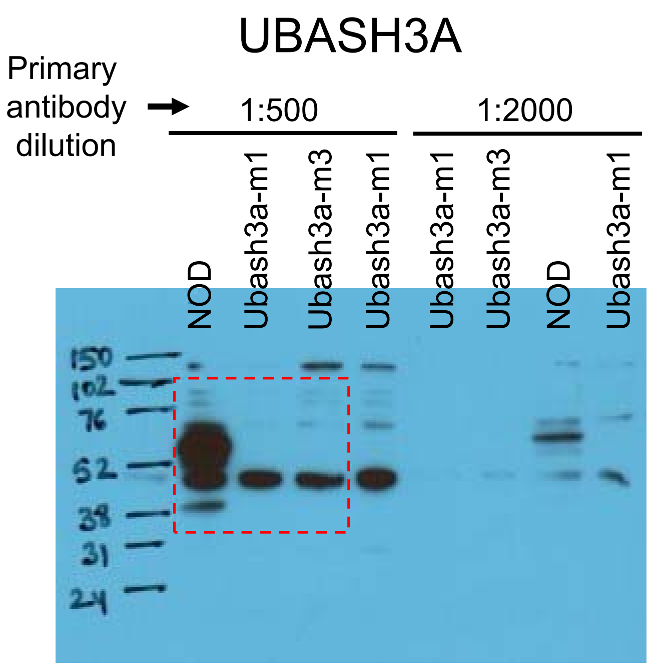

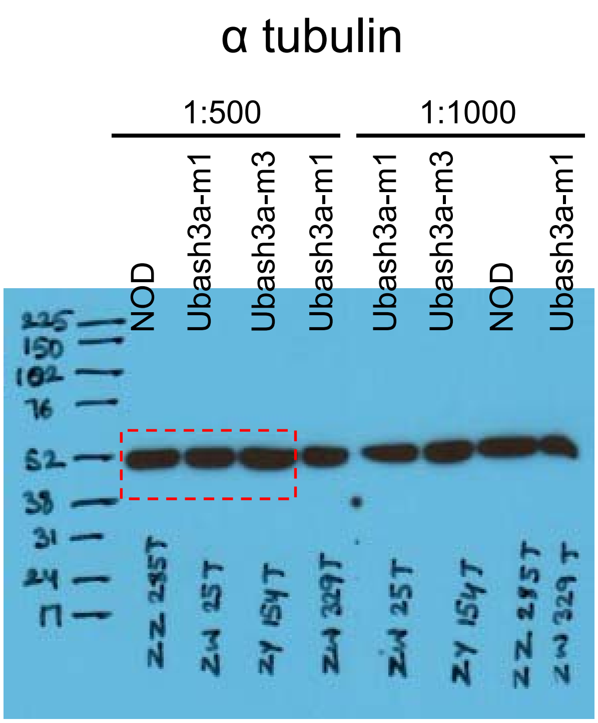

Supplementary Figure 2

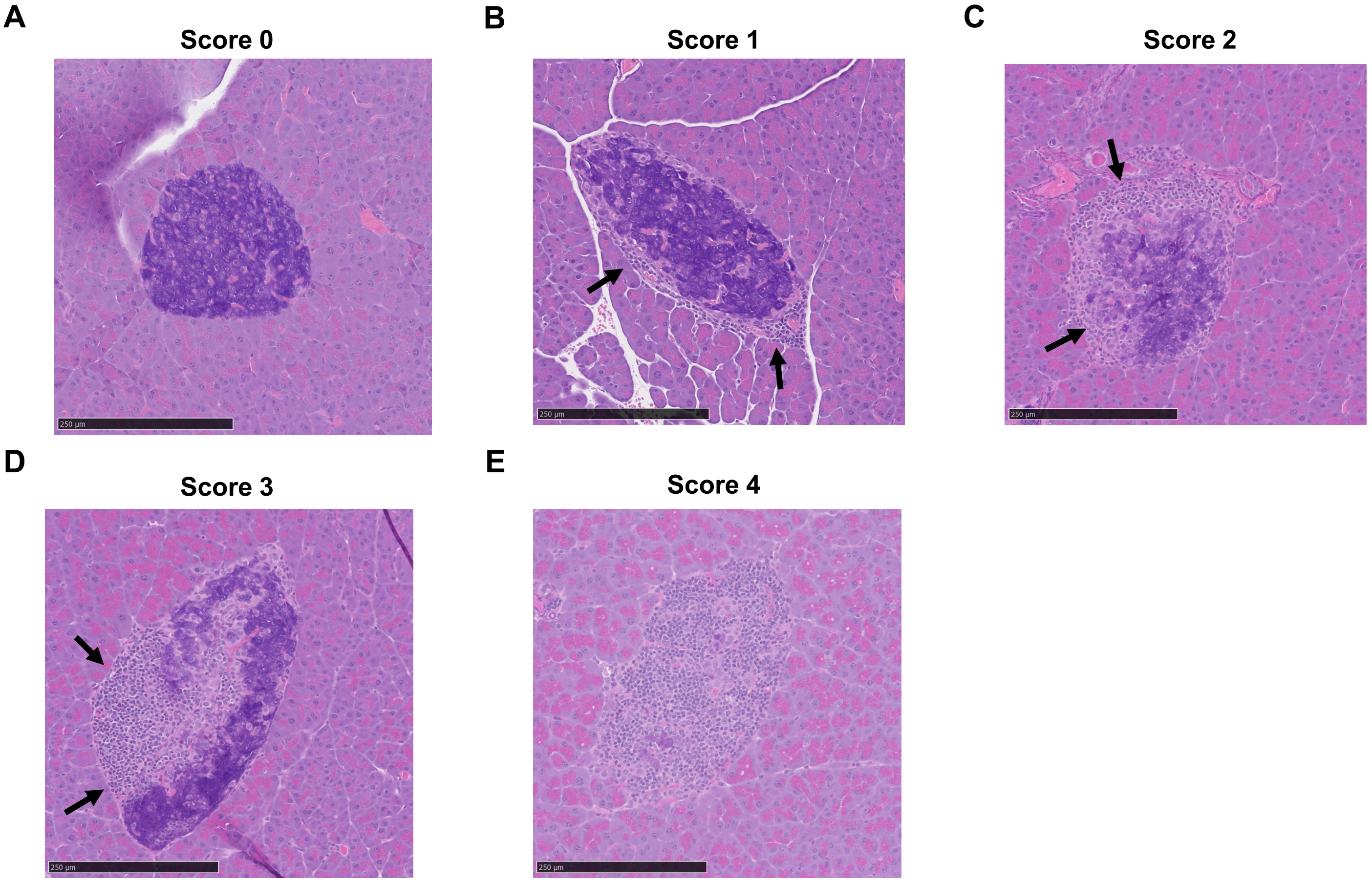

Supplementary Figure 3

A

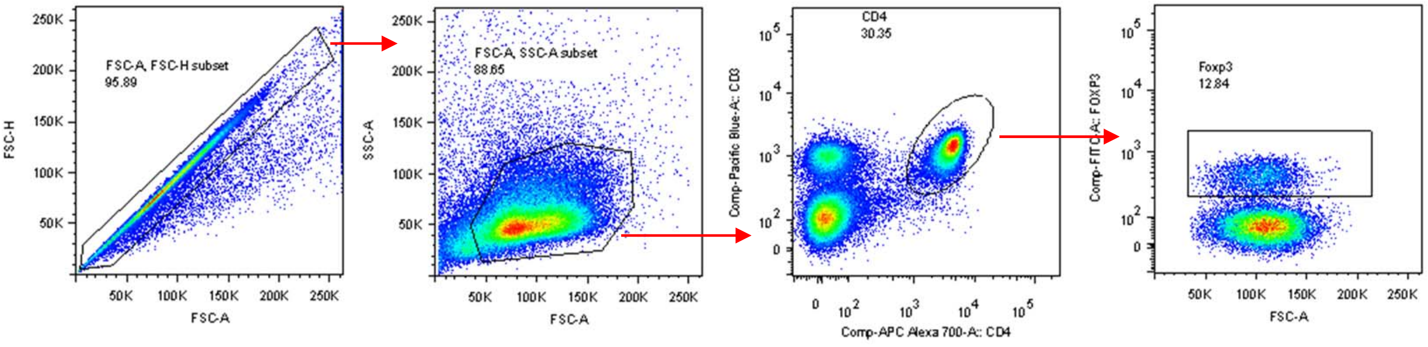

B

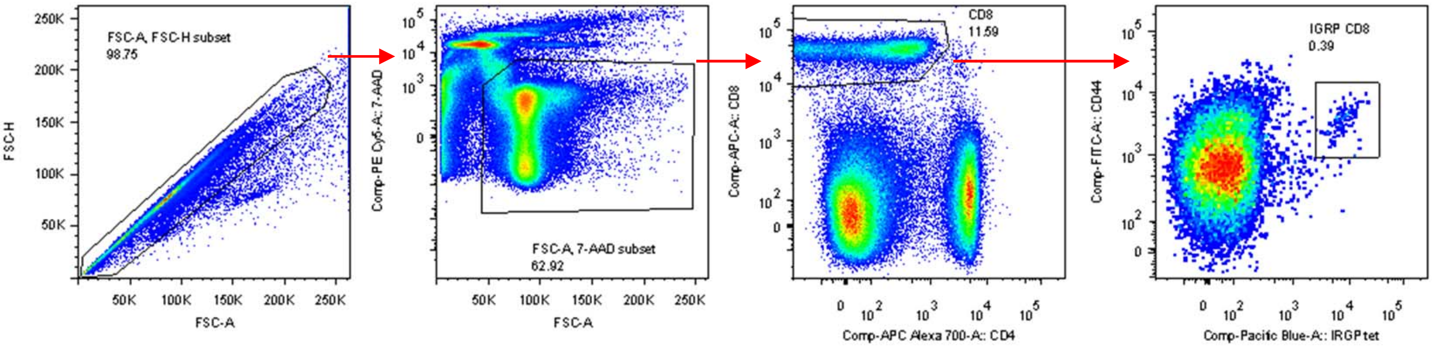

C

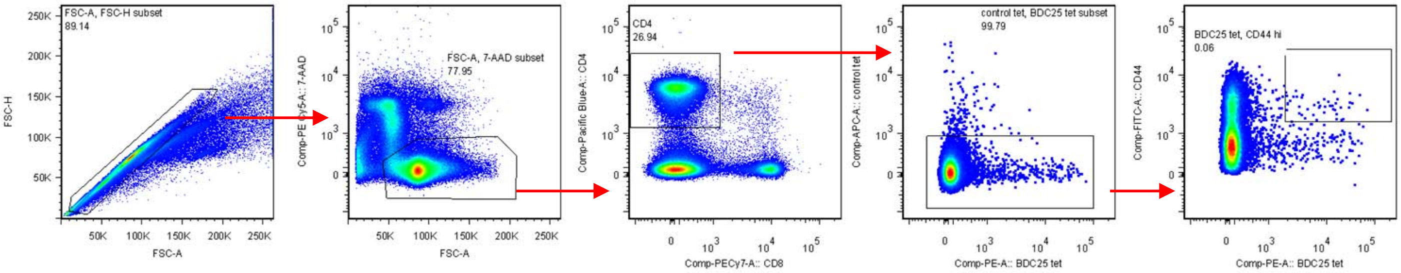

Supplement: Supplementary file 2 — Supplementary Figures [file 41598_2020_68956_MOESM2_ESM.pdf]
